# The association between self-rated health and impaired glucose tolerance in Swedish adults: A cross-sectional study

**DOI:** 10.3109/02813432.2013.784541

**Published:** 2013-06

**Authors:** Susanne Andersson, Inger Ekman, Febe Friberg, Bledar Daka, Ulf Lindblad, Charlotte A. Larsson

**Affiliations:** ^1^Institute of Health and Care Sciences, The Sahlgrenska Academy, University of Gothenburg, Gothenburg, Sweden; ^2^School of Life Sciences, University of Skövde, Sweden, Skövde, Sweden; ^3^University of Gothenburg, Centre for Person-Centred Care (GPCC); ^4^Faculty of Social Sciences, Department of Health, University of Stavanger, Norway; ^5^Institute of Medicine, Department of Primary Health Care, The Sahlgrenska Academy, University of Gothenburg, Gothenburg, Sweden; ^6^University of Lund, Department of Clinical Sciences, Malmö, Social Medicine and Global Health, Malmö, Sweden

**Keywords:** Gender, general practice, impaired glucose tolerance, self-rated health, Sweden

## Abstract

**Objective:**

To investigate gender differences in the association between self-rated health (SRH) and impaired glucose tolerance (IGT) in subjects unaware of their glucose tolerance.

**Design:**

A cross-sectional population-based study.

**Setting:**

The two municipalities of Vara and Skövde in south-western Sweden.

**Subjects:**

A total of 2502 participants (1301 women and 1201 men), aged 30–75, were randomly selected from the population.

**Main outcome measures:**

IGT was regarded as the outcome measure and SRH as the main risk factor.

**Results:**

The prevalence of IGT was significantly higher in women (11.9%) than in men (10.1%), (p = 0.029), as was the prevalence of low SRH (women: 35.4%; men: 22.1%, p = 0.006). Both men and women with low SRH had a poorer risk factor profile than those with high SRH, and a statistically significant crude association between SRH and IGT was found in both men (OR = 2.8, 95% CI 1.8–4.4) and women (OR = 1.5, 95% CI 1.0–2.2, p = 0.033). However, after controlling for several lifestyle factors and biomedical variables, the association was attenuated and remained statistically significant solely in men (OR = 2.3, 95% CI 1.2–4.3).

**Conclusion:**

The gender-specific associations found between SRH and IGT suggest that SRH may be a better indicator of IGT in men than in women. Future studies should evaluate the utility of SRH in comparison with objective health measures as a potential aid to health practitioners when deciding whether to screen for IGT and T2DM.

- People with previously unknown diabetes have lower self-rated health than those with normal glucose metabolism.
- The authors investigated whether previously unknown impaired glucose tolerance was associated with self-rated health, and whether the association differed by gender.
- Low self-rated health was independently associated with impaired glucose tolerance solely in men.

## Introduction

Type 2 diabetes mellitus (T2DM) is a common chronic disease, which substantially increases the risk of cardiovascular disease (CVD). The prevalence of T2DM is estimated to continue to increase, thus posing a serious challenge at both an individual and a societal level [1]. Impaired glucose tolerance (IGT) is associated with a six-fold risk of progression to T2DM [2], and can thus be regarded as a precursor to T2DM. However, IGT is also in itself associated with increased risk of CVD [3].

Self-rated health (SRH) is a subjective measure of health, which has been associated with increased mortality both in a general population [4,5] and in patients with T2DM [6]. Whereas SRH, as expected, is lower in patients with T2DM than in healthy individuals [7,8], low SRH has also been associated with previously unknown diabetes [8], and with development of both IGT and T2DM in longitudinal studies [9]. Although those studies did not specifically focus on gender differences, such differences have been indicated before. For example, low SRH in subjects with T2DM has been associated with an increased risk of mortality solely in men [10], and an association between psychological distress and IGT was considerably more prominent in men than in women [11]. Furthermore, a General Health Questionnaire depression subscale has been associated with incidence of diabetes and IGT in men, albeit not in women [12]. These findings suggest the need for a further examination of potential differences between genders. Thus, the present study aimed to study gender differences with regard to prevalence of IGT and SRH, and with regard to the association between SRH and IGT in a random population-based sample of individuals unaware of their glucose tolerance.

## Material and methods

### Participants

**Figure 1.**
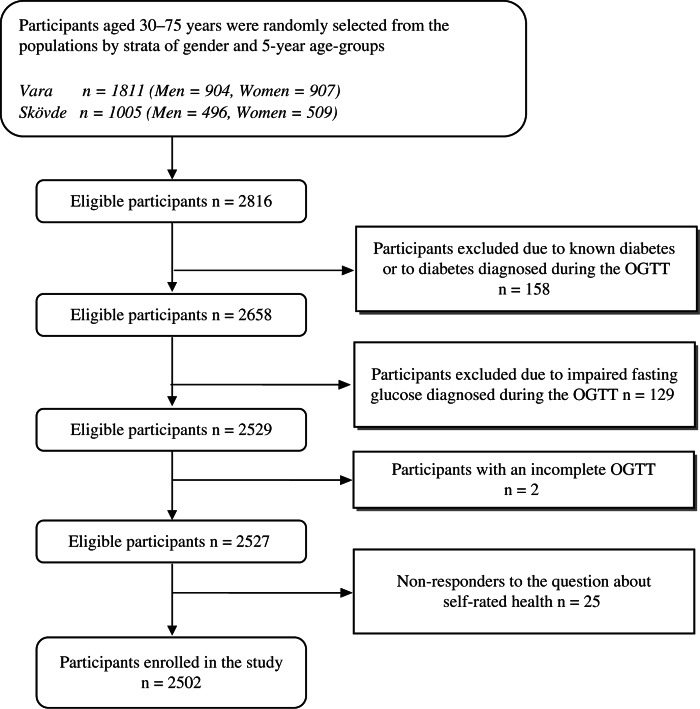
Flowchart of eligible and finally enrolled participants in the study: The Vara–Skövde cohort, Sweden, 2002–2005.

In 2002–2005, a health survey consisting of two visits to the local primary health care centre was conducted in the municipalities of Vara and Skövde, south- western Sweden, as part of the Skaraborg Project [13]. Participants aged 30–75 were randomly selected from the population by strata of gender and five-year age-groups (76% participation rate), and 2502 participants were included for the present study (Figure 1).

### Methods

As described previously in greater detail [13], data on medical history, socio-demography, SRH, and lifestyle were collected by self-administrated questionnaires on the first visit. SRH was assessed by the question: “How do you rate your current health status in general?”, with the answer alternatives “excellent”, “good”, “fair”, “poor”, and “very poor” [6,14]. These alternatives were dichotomized into high SRH (excellent + good) and low SRH (all other alternatives). Current smoking was defined as daily smoking. Leisure-time physical activity (LTPA) was measured by four alternative answers to the question: “How much physical activity do you engage in during your leisure time?” [15], and dichotomized as high and low LTPA [13]. Lack of sleep was assessed by the question “Do you feel that you get enough sleep to feel thoroughly rested?” The answers were: (1) Yes, usually; (2) Yes, but not often enough; (3) No, never or almost never. Alternatives 2 and 3 were merged for the analyses. Educational level was assessed by 10 alternatives, which were dichotomized as two levels (primary school only, versus anything above). Previous CVD was defined as a history of angina, atrial fibrillation, acute myocardial infarction, coronary heart disease, heart failure, or stroke. Previous hyperlipidaemia was defined as a physician's prescription of treatment for high serum lipids. Alcohol consumption (grams/week) was assessed by questions concerning the quantities of beer, wine, or strong liquor, respectively, consumed during the past 30 days [16].

Known diabetes was defined as a physician's diagnosis of diabetes. In participants without known diabetes, a standard oral glucose tolerance test (OGTT) was performed after a 10-hour overnight fast [17]. Blood samples in a fasting state and two hours after glucose administration were collected and analysed for plasma glucose and serum insulin. Based on the OGTT, normal glucose tolerance was defined as a fasting glucose level of ≤ 6.0 mmol/l and a two-hour level of < 7.8 mmol/l [17]. IGT was defined as a fasting glucose level of < 7.0 mmol/l and a two-hour level of 7.8–11.0 mmol/l, and impaired fasting glucose as a fasting level of 6.1–6.9 mmol/l and a two-hour level of < 7.8 mmol/l [17]. New cases of diabetes were defined as participants having a fasting glucose level of ≥ 7.0 mmol/l or a two-hour level of ≥ 11.1 mmol/l [17]. Insulin resistance was estimated by the homeostasis model assessment of insulin resistance (HOMA-ir), calculated as fasting glucose × fasting insulin/22.5 [18].

The second visit, two weeks after the first, included a physical examination with anthropometric measurements. All participants were seen by one of the two specially trained nurses who conducted all study visits. Systolic and diastolic right brachial arterial blood pressures were recorded to the nearest 2 mm Hg, in a supine position after five minutes’ rest. Body weight was measured to the nearest 0.1 kg, standing height to the nearest centimetre, and body mass index (BMI) was calculated as weight (kg)/height^2^ (m^2^).

### Statistical analyses

The statistical analyses were performed using SPSS for Windows (version 19.0), and results are presented separately for men and women. Differences between genders and IGT (high/low), respectively, were examined by general linear model for continuous variables and by logistic regression analysis for proportions. IGT was considered to be the outcome measure and thus used as the dependent variable in all analyses, whereas either gender or SRH was used as the independent variable. Confounding by age, BMI, alcohol consumption, daily smoking, educational level, HOMA-ir, fasting glucose, hypertension, previous CVD, previous hyperlipidaemia, HDL cholesterol, lack of sleep, and LTPA was assessed by stratification and by multivariate analyses. The potential confounding factors were chosen based on a theoretical model of factors previously found to be associated with both SRH and IGT. All tests were two-sided and statistical significance was accepted at p < 0.05.

## Results

**Figure 2.**
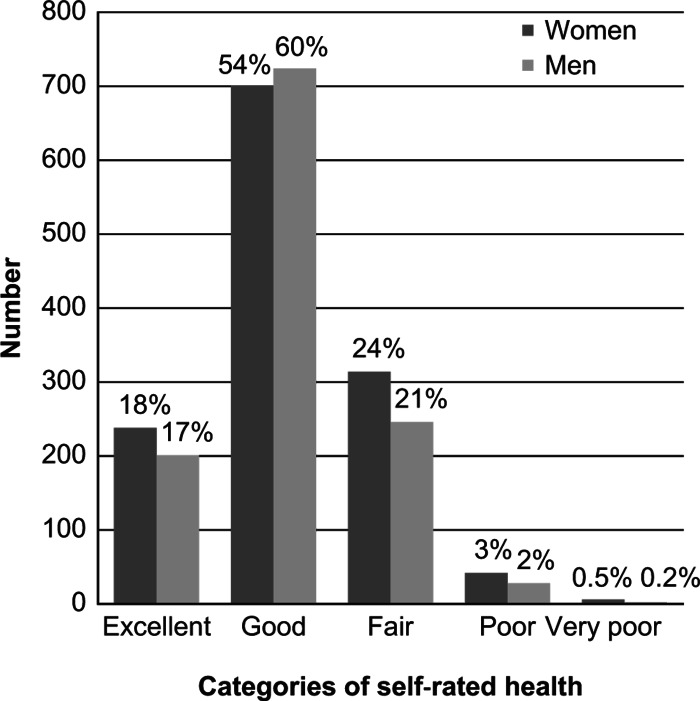
Distribution of self-rated health in men and women, respectively: The Vara–Skövde cohort, Sweden, 2002–2005.

The majority of participants gave a positive rating of their general health (Figure 2), although significantly more women than men rated their health as poor (Table I). Women also had significantly higher levels of two-hour plasma glucose and higher frequencies of IGT, and more often than men reported low LTPA and daily smoking. Men on the other hand had significantly higher levels of fasting glucose, HOMA-ir, LDL cholesterol, systolic and diastolic blood pressure, and lower levels of HDL cholesterol. Men also to a significantly higher degree than women reported higher levels of alcohol consumption, hyperlipidaemia, and previous CVD, and lower levels of education (Table I).

**Table I.** Characteristics of men and women in the Vara–Skövde cohort, Sweden, 2002–2005.

	Women		Men		
	Mean^a^	SD/CV^a^ (q1–q3)	Mean^a^	SD/CV^a^ (q1–q3)	
Characteristics	(n = 1301)		(n = 1201)		p-value
Age, years	46.8	11.2	46.5	11.2	0.470
Waist circumference, cm	84.5	11.2	93.9	11.2	< 0.001
Body mass index, kg m^–2^	26.4	4.3	26.6	4.3	0.135
Fasting p-glucose, mmol L^–1^	5.1	0.4	5.3	0.4	< 0.001
2 h p-glucose, mmol L^–1^	5.5	1.5	5.2	1.5	< 0.001
HOMA-ir	1.27	67.6	1.61	69.0	< 0.001
Systolic BP, mm Hg	118	13.7	123	13.7	< 0.001
Diastolic BP, mm Hg	68	9.4	71	9.4	< 0.001
LDL cholesterol	3.1	0.9	3.4	0.8	< 0.001
HDL cholesterol	1.40	0.32	1.21	0.31	< 0.001
Alcohol consumption g/week^b^	22.6	(0–32)	61.2	(13–78)	< 0.001^c^
	n	%	n	%	p-value
Low self-rated health	362	35.4	276	22.1	0.006
IGT	125	11.9	85	10.1	0.029
Low LTPA	889	72.7	688	60.3	< 0.001
Daily smoking	268	28.0	176	15.0	< 0.001
Lack of sleep	515	39.0	445	32.3	0.144
Primary school only	274	33.9	341	39.8	< 0.001
Previous CVD	28	3.8	42	5.7	0.023
Previous hyperlipidaemia	46	5.8	61	7.1	0.037
Hypertension	156	18.8	136	16.8	0.773

Notes: All means were adjusted for age, and all proportions were age-standardized using the Vara population as standard. P-glucose = plasma glucose; HOMA-ir = the homeostasis model assessment of insulin resistance; BP = blood pressure; IGT = impaired glucose tolerance; LTPA = leisure-time physical activity; CVD = cardiovascular disease. ^a^For alcohol consumption data are means and q1–q3 (quartile 1–quartile 3), for HOMA-ir data are geometric means (anti-log) and coefficient of variance (CV, expressed as a percentage), and for all other variables data are means and standard deviations (SD). ^b^12 g alcohol is equivalent to approximately one glass of wine (12–15 cl) or one small beer (33 cl). ^c^The p-value accounts for the generally higher physiological acceptance of alcohol in men as compared with women.

When comparing participants with high versus low SRH, both men and women with low SRH were more often smokers, had significantly higher levels of HOMA-ir and BMI, and had significantly lower levels of HDL cholesterol (Table II). Furthermore, men with low SRH also had higher fasting glucose values and more often had IGT than men with high SRH (Table II).

**Table II.** Characteristics of male and female study participants with high and low self-rated health, respectively: The Vara–Skövde cohort, Sweden, 2002–2005.

	High SRH		Low SRH		
Characteristics	Mean^a^	SD/CV^a^ (q1–q3)	Mean^a^	SD/CV^a^ (q1–q3)	p-value
Women	n = 930		n = 362		
Age, years	45.9	10.9	49.2	11.9	< 0.001
Body mass index, kg m^–2^	25.7	4.8	28.2	4.8	< 0.001
Fasting p-glucose, mmol L^–1^	5.1	0.4	5.2	0.4	0.076
HOMA-ir	1.08	66.9	1.91	65.6	< 0.001
Systolic BP, mm Hg	118	14.1	119	14.1	0.103
Diastolic BP, mm Hg	68	9.4	69	9.5	0.395
LDL cholesterol	3.1	0.8	3.2	0.8	0.087
HDL cholesterol	1.43	0.34	1.35	0.34	< 0.001
Alcohol consumption g/week^b^	24.1	(2–34)	18.5	(0–25)	0.005^c^
	n	%	n	%	p-value
IGT	80	8.5	45	12.4	0.221
Low LTPA	598	65.5	291	83.9	< 0.001
Daily smoking	177	18.8	91	25.1	0.009
Lack of sleep	297	31.8	218	60.7	< 0.001
Primary school only	157	17.1	117	33.6	< 0.001
Previous CVD	13	1.4	15	4.2	0.058
Previous hyperlipidaemia	30	3.2	16	4.4	0.983
Hypertension	93	9.9	63	17.4	0.086
Characteristics	Mean^a^	SD/CV^a^ (q1–q3)	Mean^a^	SD/CV^a^ (q1–q3)	p-value
Men	n = 925		n = 276		
Age, years	46.3	11.0	47.2	11.5	< 0.001
Body mass index, kg m^–2^	26.4	3.4	27.4	3.4	< 0.001
Fasting p-glucose, mmol L^–1^	5.3	0.4	5.4	0.4	0.045
HOMA-ir	1.51	67.5	1.98	67.0	0.004
Systolic BP, mm Hg	122	13.0	123	13.1	0.599
Diastolic BP, mm Hg	71	9.4	72	9.4	0.586
LDL cholesterol	3.4	0.9	3.4	0.9	0.573
HDL cholesterol	1.22	0.27	1.17	0.28	0.008
Alcohol consumption g/week^b^	60.0	(15–80)	64.9	(8–76)	0.498^c^
	n	%	n	%	p-value
IGT	48	5.2	37	13.4	< 0.001
Low LTPA	482	54.0	206	76.3	< 0.001
Daily smoking	120	13.0	56	20.3	0.003
Lack of sleep	274	29.7	171	62.6	< 0.001
Primary school only	249	27.3	92	34.5	0.007
Previous CVD	26	2.8	16	5.8	0.043
Previous hyperlipidaemia	40	4.3	21	7.6	0.053
Hypertension	101	10.9	35	12.7	0.714

Notes: A general linear model was used to calculate differences in means of continuous variables between participants reporting low self-rated health and those reporting high self-rated health. Logistic regression was used to estimate associations between categorical variables. All analyses were adjusted for differences in age distribution. P-glucose = plasma glucose; HOMA-ir = the homeostasis model assessment of insulin resistance; BP = blood pressure; IGT = impaired glucose tolerance; LTPA = leisure-time physical activity; CVD = cardiovascular disease. ^a^For alcohol consumption data are means and q1–q3 (quartile 1–quartile 3), for HOMA-ir data are geometric means (anti-log) and coefficient of variance (CV, expressed as a percentage), and for all other variables data are means and standard deviations (SD). ^b^12 g alcohol is equivalent to approximately one glass of wine (12–15 cl) or one small beer (33 cl). ^c^The p-value accounts for the generally higher physiological acceptance of alcohol in men as compared with women.

When the association between SRH and IGT was further explored in logistic regression analyses, a statistically significant association between SRH and IGT was revealed in both men and women in the crude model (Table III). In men, this association was stronger and only slightly attenuated after adjustments for lifestyle and several biomedical risk factors (OR full model 2.3, 95% CI 1.2–4.3). In women on the other hand, the association was no longer significant after adjustments for any factor, with BMI as the strongest confounder (Table III).

**Table III.** Association between self-rated health and impaired glucose tolerance in women and men, respectively: The Vara–Skövde Cohort, Sweden, 2002–2005.

	Women		Men	
Adjustments	OR	95 % CI	OR	95 % CI
Crude	1.5	1.0–2.2*	2.8	1.8–4.4
Age	1.3	0.9–1.9	2.8	1.8–4.5
Age and body mass index	1.0	0.6–1.5	2.5	1.6–4.1
Age and fasting p-glucose	1.1	0.7–1.7	2.7	1.6–4.6
Age and HOMA-ir	1.0	0.6–1.5	2.7	1.6–4.4
Age and HDL cholesterol	1.1	0.8–1.7	2.6	1.6–4.2
Age and alcohol consumption	1.3	0.8–1.9	2.9	1.8–4.7
Age and hypertension	1.2	0.8–1.8	2.9	1.8–4.6
Age and LTPA	1.2	0.8–1.8	2.4	1.5–3.9
Age and daily smoking	1.3	0.9–2.0	2.9	1.8–4.6
Age and lack of sleep	1.3	0.8–1.9	2.5	1.5–4.2
Age and educational level	1.4	0.9–2.1	2.8	1.7–4.6
Age and previous hyperlipidaemia	1.3	0.8–1.8	2.8	1.7–4.5
Age and previous CVD	1.2	0.8–1.9	2.7	1.7–4.4
All above	1.0	0.6–1.7	2.3	1.2–4.3

Notes: Associations between self-rated health (independent variable, low versus high) and impaired glucose tolerance (dependent variable) were analysed using logistic regression analysis and expressed as odds ratios (OR) with 95% confidence intervals (95% CI). Age, body mass index, fasting glucose, HOMA-ir (the homeostasis model assessment of insulin resistance), and HDL cholesterol were all entered into the model as continuous variables, whereas the rest of the variables were dichotomized. Missing values: body mass index: 9 women and 5 men; previous hyperlipidaemia: 1 man; previous CVD: 2 women; level of education: 34 women and 21 men; leisure-time physical activity (LTPA): 41 women and 38 men; lack of sleep: 9 women and 5 men; alcohol consumption: 39 women and 31 men; daily smoking: 2 women and 6 men; HOMA-ir: 7 women and 7 men. *p = 0.033.

## Discussion

### Principal findings

Both men and women with poor SRH had a more atherogenic risk-factor profile than participants with high SRH. In a crude model, a statistically significant inverse association between SRH and IGT was seen in both men and women. However, after adjustments for confounders the association remained solely in men.

### Strengths and weaknesses of the study

The large random, population-based sample and the high participation rates all strengthen the generalizability of the current results. The validity is also supported by the fact that two specially trained registered nurses performed all study visits. Furthermore, all participants without previously known diabetes were given an OGTT to systematically identify those with IGT, using current standard definitions [17]. All questionnaires were completed before the results of the OGTT were revealed, and SRH has previously been shown to predict e.g. mortality [4–6,10] and IGT/T2DM [9]. Several potential confounders were also addressed; however, dietary habits were not, and inadequate diet is associated both with SRH and with the development of IGT. However, the effect of diet on IGT and SRH, respectively, is probably mediated mainly through obesity, which was adjusted for in the present study. With regard to the self-reported data, the possibility of misclassification due to misreporting must be considered. Whereas misclassification of LTPA is possible, the instrument used here has shown good internal validity both in the current population [13] and elsewhere [15]. However, under-reporting of alcohol consumption is common [19] and might have affected the results. Finally, as the study is cross-sectional, causality cannot be established.

### Relation to other studies

Regarding the association seen here between SRH and IGT, comparative studies are scarce. In an Australian cohort the association between SRH and IGT was explained by confounding in cross-sectional analyses [8], whereas poor SRH at baseline was independently associated with an increased incidence of both IGT and T2DM at follow-up [9]. However, the latter results are not supported by Dankner et al. [7] who found no association between SRH and newly diagnosed T2DM. Still, as their participants were older, and older subjects generally rate their health as poorer than younger ones [20], any potential association with SRH might have been diminished.

Although the Australian studies [8,9] included both men and women, they did not specifically focus on gender differences and only adjusted their results for gender. In contrast, all the analyses in the current study were gender-specific and revealed an independent association between SRH and IGT solely in men, whereas the crude association in women was a confounding effect mainly caused by differences in BMI. A closer link between bodyweight and SRH in women than in men is supported by previous studies [14,21,22]. Furthermore, studies on psychological distress and IGT [11], depression and IGT/T2DM [12], and SRH and mortality in subjects with T2DM [6,10] have found associations to be stronger, or solely present in men. Taken together, this indicates that other mechanisms may be involved in men. Although different coping strategies seem plausible, this is not supported here with regard to lifestyle-related factors, as such factors did not substantially influence the results in men. However, the lower frequency of men reporting poor SRH, as seen here and elsewhere [10,20], might be a reflection of under-reporting of physiological distress in men, as they are known to report somatic symptoms to a lesser extent than women do [23], and to be more reluctant to seek medical advice [24]. As suggested by Eriksson et al. [11], these factors might have strengthened the association between SRH and IGT in men. Thus, different forms of stress have been linked to the development of insulin resistance through activation of the neuroendocrine stress system [25], and such activation is more likely to have occurred in men by the time they finally report any symptoms. Moreover, higher prevalence of IGT in women, seen here and elsewhere [4,26,27], has in one study [27] been suggested to be a consequence of using a fixed glucose load in the OGTT, as the gender difference observed there disappeared after adjustment for body height. Although an exploration of this in further detail was beyond the scope of the present study, adjustment for body height did not affect the main findings here with regard to SRH and IGT (data not shown).

The possibility of the current results reflecting an effect of IGT on SRH rather than the contrary must also be acknowledged. A qualitative study [28] within the same cohort as the present study has previously found subjects with IGT to experience diabetes- related symptoms, such as fatigue, frequent urination, and thirst. Although qualitative studies are not designed to explore differences on a group level, one might speculate that men with IGT to a greater extent than women might be aware of such symptoms and as a consequence report poorer SRH. However, this is not supported by the findings in a recent study in subjects with T2DM [29], where women reported more diabetes-related symptoms than men.

### Conclusions and implications for clinicians and future research

Identifying subjects with IGT and T2DM early on is important to facilitate lifestyle or medical interventions in order to treat manifest T2DM or prevent the development from IGT to T2DM. The independent association found here between SRH and IGT in men suggests that, at least in men, SRH might be a useful indicator to consider when health practitioners make decisions about screening for IGT and T2DM. Thus, future studies should evaluate the utility of SRH in comparison with objective health measures. Moreover, as no association between SRH and IGT was found in women in the present study, future studies should also further explore the relationships between gender-specific factors and IGT.
